# Environmental sustainability in urologic practices: a systematic review

**DOI:** 10.1007/s00345-025-05522-7

**Published:** 2025-03-06

**Authors:** A. Tozsin, A. Aydin, S. Silay, A. E. Demet, T. Knoll, T. Herrmann, M. De Bruin, P. Dasgupta, J. Rassweiler, Selcuk Guven, K. Ahmed

**Affiliations:** 1 Department of Urology, Trakya University School of Medicine Hospital, Edirne, Turkey; 2 Faculty of Life Sciences and Medicine, King’s College London, Guy’s and St. Thomas’ NHS Foundation Trust, King’s Health Partners, London, UK; 3 Istanbul Medipol University, Istanbul, Turkey; 4 Department of Energy Systems Engineering, Necmettin Erbakan University, Konya, Turkey; 5 Klinikum Sindelfingen-Boeblingen, University Medicine Mannheim, Sindelfingen, Germany; 6 Department of Urology, Kantonspital Thurgau AG Pfaffenholzstrasse, Frauenfeld, Switzerland; 7 Department of Urology, Biomedical Engineering and Physics, University of Amsterdam, Amsterdam, The Netherlands; 8 Department of Urology and Andrology, Danube Private University, Krems, Austria; 9 Department of Urology, Necmettin Erbakan University, School of Medicine, Konya, Turkey; 10 MRC Centre for Transplantation, King’s College London, London, UK; 11 Sheikh Khalifa Medical City, Abu Dhabi, UAE; 12 Khalifa University, Abu Dhabi, UAE

**Keywords:** Carbon footprint, Environmental impact, Greenhouse gas, Sustainability

## Abstract

**Purpose:**

The aim of this systematic review is to assess the environmental impact of urologic procedures and equipment (P), specifically comparing emissions and waste generation between single-use and reusable devices (I and C), while also exploring strategies for emission reduction and providing relevant recommendations for sustainable practices in urology.

**Methods:**

The review registered to PROSPERO (ID: CRD42024576865) and adhered to the Preferred Reporting Items for Systematic Reviews and Meta-analyses (PRISMA) guidelines. A systematic search was conducted to identify studies addressing sustainability, carbon footprint, and environmental impact in urology. A total of 7714 records were initially identified, of which ten met the inclusion criteria. Study quality was assessed using the QUADAS scoring system to evaluate risk of bias and applicability concerns.

**Results:**

Ten studies met the inclusion criteria, focusing on the environmental impacts of urologic devices and procedures (O). Single-use cystoscopes demonstrated lower carbon dioxide (CO_2_) emissions per procedure (2.41 kg) compared to their reusable counterparts (4.23 kg) but produced more waste (622 g). Reusable cystoscopes, while having a lower cumulative waste per-use, increased emissions due to energy-intensive reprocessing. For ureteroscopes, single-use devices generated less CO_2_ but significantly more solid waste. TURBT procedures had a high carbon footprint (131.8 kg CO_2_ per procedure), largely from single-use items and sterilization. Robotic prostatectomy produced a lower carbon footprint (47,313 g CO_2_) than laparoscopic methods, emphasizing the potential for energy-efficient techniques to reduce emissions in urology.

**Conclusion:**

A hybrid approach in urology, focusing on improving sterilization processes and developing eco-friendly single-use alternatives, may provide a balanced approach toward sustainability.

**Supplementary Information:**

The online version contains supplementary material available at 10.1007/s00345-025-05522-7.

## Introduction

The healthcare sector contributes to approximately nearly 5% of global greenhouse gas emissions, making it a significant player in the global climate crisis [1]. Sustainability has become a significant concern in healthcare, particularly in specialized fields such as urology. Advancements in technology and procedural requirements in urology have resulted in significant impact upon the environment. Urological practices, particularly those involving energy-intensive procedures like cystoscopy and ureteroscopy, contribute to this carbon footprint through the extensive use of disposable instruments and the energy required for sterilization [2]. There is an increasing demand for implementing sustainable practices in the field of urology, which involves reducing dependence on disposable devices and prioritizing more environmentally friendly solutions [3, 4].

One of the main topics in urology involves the ongoing debate between single-use and reusable instruments. While single-use devices eliminate the need for reprocessing, they generate significant waste and can have a fourfold higher carbon footprint compared to their reusable counterparts per case [2, 5]. On the other hand, reusable devices, while more sustainable in terms of waste reduction, require energy-intensive sterilization processes, contributing to their overall environmental impact [3]. Studies comparing the environmental impact of these devices highlight the need for a balanced approach that considers both patient safety and environmental sustainability [6]. The transition to sustainable urological practices also includes utilizing alternative energy sources in the operating rooms and integrating environmental considerations into clinical guidelines to reduce the overall carbon footprint in healthcare [4].

This systematic review aims to evaluate the impact of urological equipment and processes on the environment through carbon dioxide (CO_2_) emissions, waste production, and carbon footprints associated with equipment usage. Additionally, the review aims to explore emission reduction strategies and provide relevant recommendations.

## Methodology

### Search strategy

**Fig. 1:**
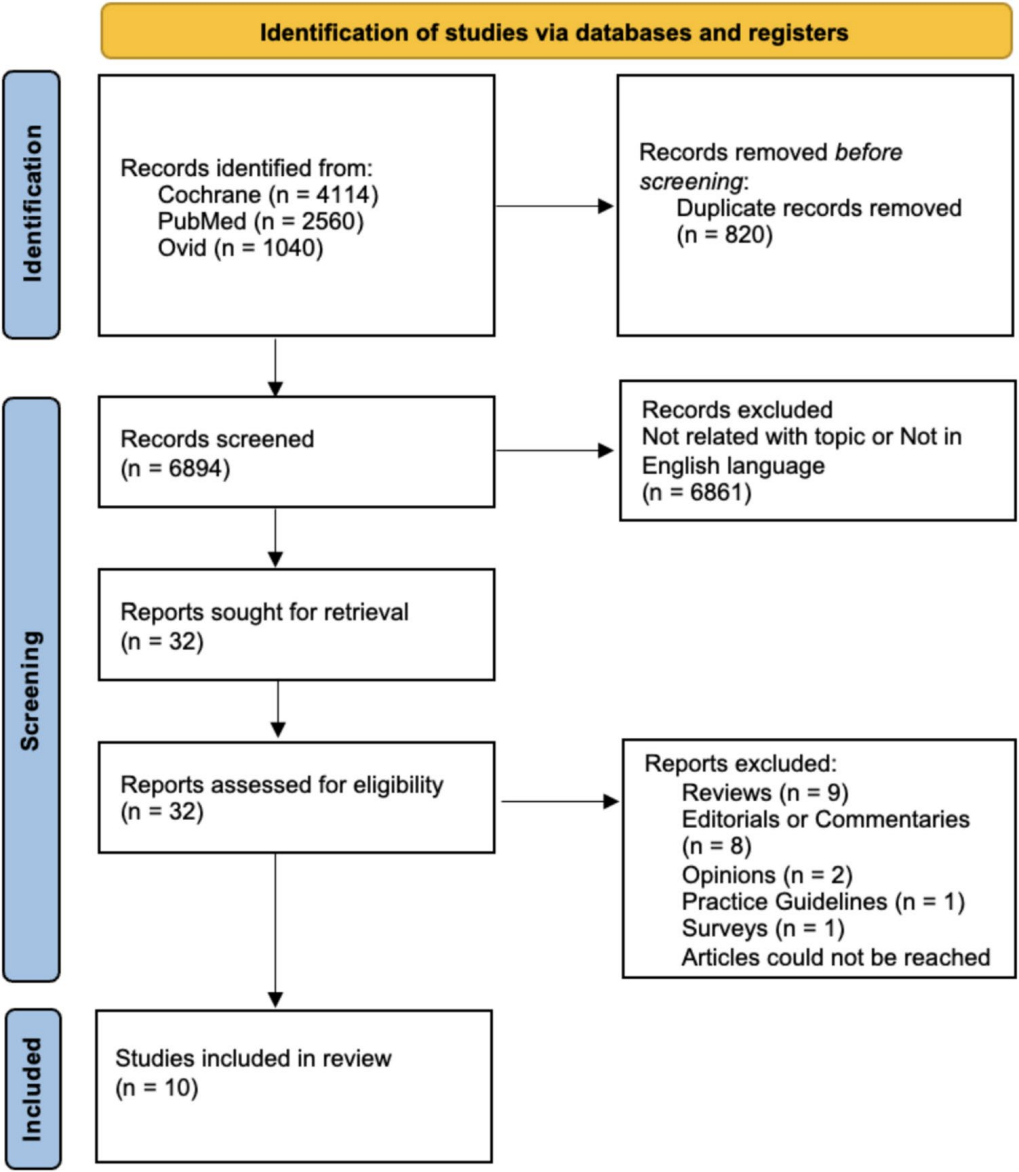
The flowchart of the systematic review according to PRISMA guidelines

This review (PROSPERO ID: CRD42024576865) followed the Preferred Reporting Items for Systematic Reviews and Meta-Analysis (PRISMA) statement (Fig. 1). A systematic literature search was conducted in the Cochrane, PubMed and Ovid databases to find studies evaluating the sustainability, carbon footprint, and environmental impacts associated with urology, with no time constraints up to November 20, 2024. Articles in the English were reviewed retrospectively using MeSH Terms. The search strategy combined terms related to environmental sustainability, carbon emissions, and urology and can be found in Supplement 1. Boolean operators were used to combine these search terms to ensure a comprehensive yet relevant literature search. The search was limited to articles in English, and results were sorted by publication date to prioritize recent research. After initial screening of titles and abstracts, full-text articles were retrieved and assessed for inclusion based on their relevance to the study objectives.

### Study selection and data extraction

Initially, 7714 records were identified from the Cochrane, PubMed and Ovid databases. After duplicates removal 6894 publications were screened. The screening process yielded 6861 records which were excluded due to irrelevance to the study's focus or not in English language. Thirty-two full-text reports were then assessed for eligibility. Reviews, editorials, commentaries, opinions, practice guidelines and surveys were excluded. Finally, ten studies met the inclusion criteria and were included in the final systematic review. Each study was reviewed by a minimum of two authors, who independently conducted screening of both the titles and abstracts, as well as full-text evaluation. In instances of disagreement, a third author conducted an additional assessment of the data. Data extraction was conducted by two authors for each article. A database was created including the following items: study design, country/year, equipment, variables of interest, CO_2_ emissions considered, waste disposal, solid waste produced, carbon footprints of waste products, sterilization method, and emissions reduction strategies.

### Study qualification

**Fig. 2:**
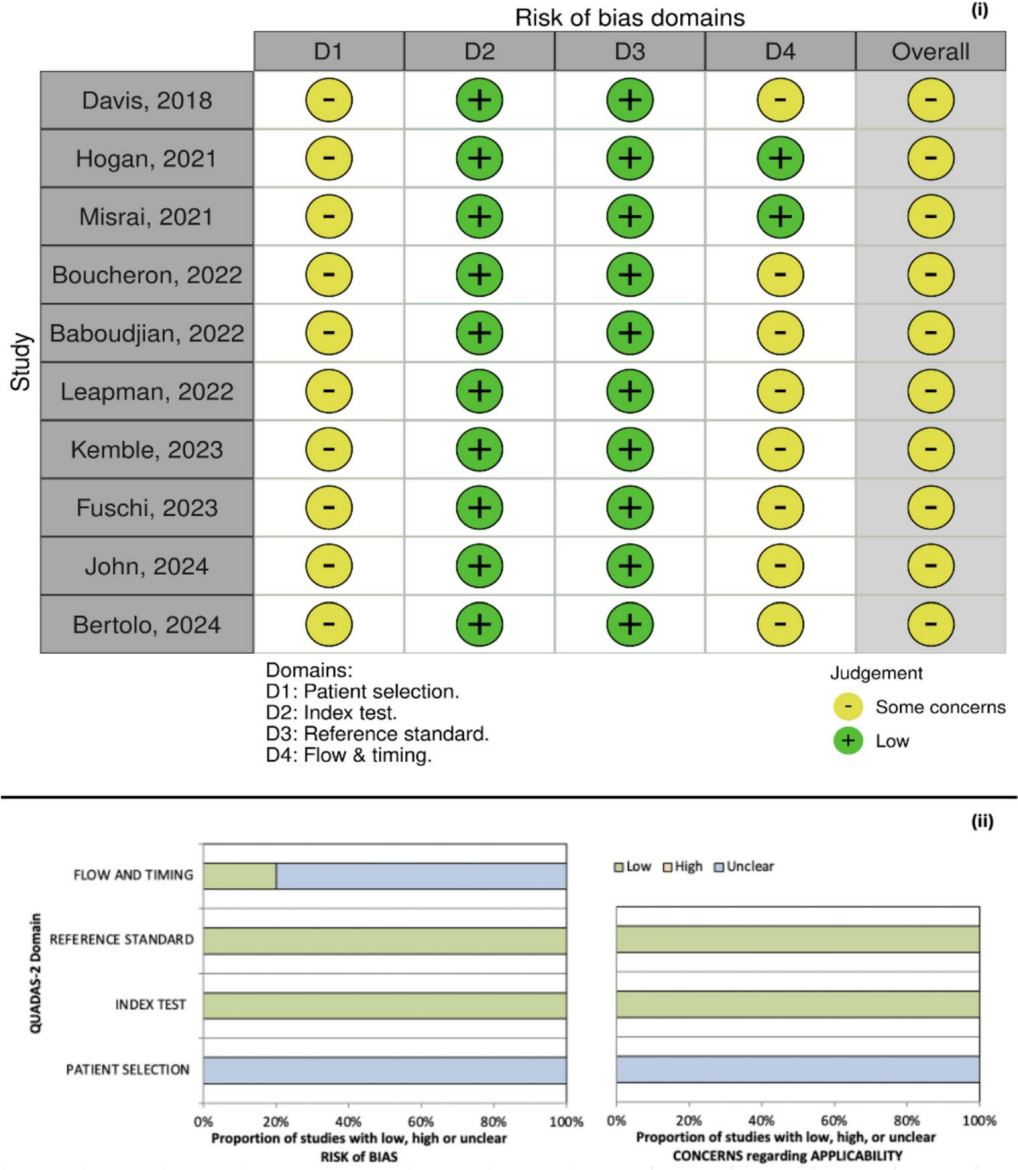
Quadas scoring (**i**) of the studies analyzed and proportion of studies with risk of bias and concerns regarding applicability (**ii**)

Study qualifications were evaluated using the Quadas scoring system (Fig. 2) to determine the risk of bias and concerns regarding applicability in each study.

## Results

### Carbon footprint and environmental impact

The impact of urological interventions or instruments on the environment were evaluated using the following outcomes: CO_2_ emissions, waste disposal, solid waste produced, carbon footprints of waste products to evaluate environmental impact. Table 1 summarizes all the studies according to CO_2_ emissions and wastes in urological procedures.

**Table 1:** Summary of studies on CO_2_ emissions and waste in urological procedures

Study	Study design	Country/year	Equipment	Variables of interests	CO_2_ emissions	Waste disposal	Solid waste produced	Carbon footprints of waste products	Sterilization method	Emission reduction strategies
Davis et al. [11]	Life cycle assessment comparative study	Australia/2018	LithovueTM single-use ureteroscope Olympus URV-F reusable ureteroscope	Manufacturing, usage, repairs, disposal, solid waste, energy consumption	Manufacturing: 11.49 kg CO_2_/kg, materials: plastic 69 kg CO_2_/kg, rubber 1.16 kg CO_2_/kg, steel 1.81 kg CO_2_/kg, electronics 150 kg CO_2_/kg	Incineration: 1.23 kg CO_2_/kg Landfill: 0.85 kg CO_2_/kg	Single-use: 0.3 kg per case Reusable: 0.06 kg per case	Single-use: 4.43 kg CO_2_/case Reusable: 4.47 kg CO_2_/case	Single-use: ethylene oxide Reusable: Olympus ETD4 washer (7.89 kg CO_2_/cycle)	Resource efficiency, recycling protocols, low-carbon energy
Hogan et al. [7]	Prospective cohort study	Ireland, Australia/2021	aScope 4 Cysto single-use CYF-VA2 reusable cystoscope	Packaging weights, solid waste converted to CO_2_ mass	Manufacturing: 8.51 kg CO_2_/kg Sterilization: 3.5 kg CO_2_/case Transport: 0.049 kg CO_2_/cystoscope	Incineration: 1.23 kg CO_2_/kg Landfill: 0.85 kg CO_2_/kg	Single-use: 622 g Reusable: 671.5 g	Single-use: 2.41 kg CO_2_/case Reusable: 4.23 kg CO_2_/case	Single-use: ethylene oxide Reusable: Olympus ETD-Double machine (3.5 kg CO_2_/case)	Resource efficiency, recycling protocols, low-carbon energy
Misrai et al. [14]	Life cycle assessment study	France/2021	Various minimally invasive surgical devices (MISDs)	Lifecycle assessment, data collection, emission factors	Estimated CO_2_: 0.07 to 3.3 kg CO_2_e/device Plastic: 2.38 kg CO_2_e/kg	Not detailed	Disposable: 3 to 584 g Packaging: Plastic and cardboard	Rezūm: 2288 grCO_2_e Plasma Loop: 1204 grCO_2_e Greenlight laser fiber: 387 gr CO_2_e UroLift: 545 grCO_2_e iTIND: 76 grCO_2_e HoLEP**:** 235 grCO_2_e	Not detailed	Transparency, material reduction, recycling, low-carbon materials, minimize material usage
Boucheron et al. [8]	Retrospective observational study	France/2022	Reusable flexible cystoscopes Disposable flexible cystoscopes (Ambu aScope S4C)	Costs, environmental impacts, micro-costing approach	Single-use: CO_2_ emissions during manufacturing Transport: CO_2_ emissions from transport	Incineration Landfill	Single-use: 200 g per procedure Reusable: 800 g per procedure	Single-use: not detailed Reusable: high reprocessing emissions (CO_2_, water consumption)	Reusable: peracetic acid high-level disinfection	Adopt disposable devices, optimize sterilization, lifecycle assessments, decarbonize supply chain
Baboudjian et al. [9]	Life cycle assessment study	France/2022	Reusable flexible cystoscopes Single-use (Ambu aScope 4 Cysto)	Lifecycle assessment, reprocessing with peracetic acid	Single-use: 2.06 kg CO_2_ eq./scope Reusable: 3.08 kg CO_2_ eq./use	Incineration	Single-use: Complete device disposal Reusable: reprocessing waste	Single-use: Lower overall environmental impacts compared to reusable	Reusable: High-level disinfection with peracetic acid	Use disposable cystoscopes, improve reprocessing, lifecycle analysis, decarbonize supply chain
Leapman et al. [13]	Life cycle assessment study	USA/2022	Prostate biopsy pathway equipment (MRI, TRUS biopsy, pathology)	Lifecycle assessment, energy use, travel, supply production	Total: 80.7 kg CO_2_e per biopsy (MRI: 42.7 kg CO_2_e, Biopsy: 33.3 kg CO_2_e, Pathology: 4.8 kg CO_2_e)	Incineration: 0.3% Landfilling: 1.7% Recycling: 1.7%	1.6 kg per biopsy	Breakdown by category: MRI, Biopsy, Pathology	Standard sterilization (autoclaves, chemical disinfectants)	Avoid unnecessary biopsies, use MRI triage, optimize sterilization
Kemble et al. [5]	Life cycle assessment study	USA/2023	Ambu aScope 4 Cysto (single-use) Olympus CYF-V2 (reusable)	Performance data from high-volume practice, repairs, decommissioning	Single-use: 1.37 kg CO_2_/case Reusable: 0.0017 kg CO_2_/case	Incineration: single-use: 0.31 kg CO_2_ Reusable: 0.000145 kg CO_2_/case	Single-use: 0.16 kg/device Reusable: 0.57 kg/device over 3920 uses	Single-use: 2.40 kg CO_2_/case Reusable: 0.53 kg CO_2_/case using Medivators	Single-use: ethylene oxide Reusable: Medivators Advantage Plus reprocessor (0.20 kg CO_2_)	Use reusable cystoscopes, optimize reprocessing, minimize packaging, adopt hybrid models
Fuschi et al. [15]	Prospective comparative study	Italy/2023	Robot Xi Da Vinci system 3D laparoscopic system by Karl Storz	Prospective data from 223 patients, materials, energy use, hospital stay	Robotic: 9506.18 g CO_2_ Laparoscopic: 12,946.73 g CO_2_	CO_2_ emissions from disposal of single-use devices, decontamination of reusable components	Laparoscopic: 1733.45 g (plastics: 1398.2 g) Robotic: 1737.41 g (plastics: 1277.20 g)	Laparoscopic: 59,674.96 g CO_2_/procedure robotic: 47,313.414 g CO_2_/procedure	Not detailed	Use reusable instruments, shorter surgical/hospital stays, energy efficiency measures
John et al. [12]	Life cycle assessment study	UK/2024	TURBT equipment Anesthesia PPE Reusable items	Inventory of materials and energy flows, patient and staff travel	Major contributors: surgical equipment, travel, gas, electricity	Incineration for single-use items, recycling for certain waste streams	Single-use surgical equipment and consumables, waste from reprocessing reusable items	Median perioperative TURBT carbon footprint: 131.8 kg CO_2_e	Reusable items processed through HSDU, including autoclave sterilization	Minimize patient travel, rationalize equipment use, optimize operating theatre lists, avoid postoperative catheterization
Bertolo et al. [10]	Retrospective micro-costing analysis	Italy/2024	Reusable Cystoscopes: Storz and Olympus brands Single-use Cystoscopes: Not specified	Data from 2022: records, observations, expert opinions. Depreciation: 5 years (reusables), 8 years (reprocessors). Sensitivity analyses included	15 kg CO_2_ saved per single-use procedure. Includes CO_2_ from reprocessing and repairs of reusables	Reusables: waste from reprocessing. Single-use: direct disposal	Reusables: 6 kg per procedure. Single-use: 3.5 kg per procedure	Reusables: higher due to reprocessing and repairs Single-use: lower, saving 15 kg CO_2_ each	Reusables: plasma gas or ethylene oxide reprocessing. Single-use: no reprocessing needed	Single-use cystoscopes save water and CO_2_. Improve efficiency by reducing waiting lists and increasing procedure frequency

#### CO_2_ emissions

When assessing CO_2_ emissions, significant differences were observed between single-use and reusable cystoscopes (Table 2). A study by Hogan et al. analyzed the single-use aScope™ 4 Cysto and reusable CYF-VA2 cystoscope, finding that single-use devices emitted a lower carbon footprint per procedure at 2.41 kg CO_2_, compared to 4.23 kg CO_2_ for the reusable alternative. This difference is primarily due to the energy requirements of reprocessing reusable cystoscopes, which demand 10.5 kW of electricity per cycle, adding approximately 3.5 kg CO_2_ per use. For single-use devices, manufacturing accounted for 1.34 kg CO_2_ emissions, with waste incineration contributing an additional 0.61 kg CO_2_ per procedure [7]. Boucheron et al. reported that the environmental impact of cystoscopy varies significantly by device type. Reusable cystoscopes generate more waste and consume large amounts of water, requiring 60 L per procedure for reprocessing. Although single-use devices eliminate this reprocessing stage and do not require water, their manufacturing process yields higher CO_2_ emissions [8]. Further analysis by Baboudjian and colleagues estimated, using Simapro software, that single-use cystoscopes emit 2.06 kg CO_2_ equivalent per device, while reusable cystoscopes emit 3.08 kg CO_2_ equivalent per use, with the emissions disparity largely attributable to the energy and resources needed for reprocessing [9]. Kemble et al. found the single-use Ambu aScope™ 4 Cysto produced 2.40 kg CO_2_ per procedure, mainly from manufacturing (1.59 kg CO_2_), while the reusable Olympus CYF-V2 showed a lower footprint of 0.53 kg CO_2_ using the Medivators Advantage Plus™ system, though this increased to 1.04 kg CO_2_ when the more energy-intensive ASP Evotech^®^ ECR system was used [5]. An economic and environmental assessment by Bertolo et al. concluded that single-use cystoscopes offer considerable CO_2_ savings of 15 kg per procedure compared to reusables, with projected annual savings of €133,146.49. Single-use devices reduced waste by 2965 kg and lowered CO_2_ emissions (CO_2_e) by 17,790 kg annually, although reusable devices generated more waste during reprocessing, estimated at 6 kg per procedure, compared to 3.5 kg for single-use instruments due to sterilization consumables [10]. In ureteroscopy, Davis et al. evaluated the environmental impact of single-use and reusable ureteroscopes, finding that the single-use Lithovue™ had a carbon footprint of 4.43 kg CO_2_ per procedure, primarily from manufacturing (3.45 kg CO_2_), while the reusable Olympus URV-F produced slightly higher emissions at 4.47 kg CO_2_ per use. The increased footprint was largely due to the energy-intensive sterilization process, requiring 9.2 kWh per cycle, resulting in 7.89 kg CO_2_ emissions [11]. John et al. analyzed the environmental impact of trans-urethral resection of bladder tumor (TURBT) procedures. The study revealed a median carbon footprint of 131.8 kg CO_2_e per procedure. A significant portion, 57%, of the emissions originated from the manufacturing and transport of goods, with contributions from surgical equipment, staff travel, and sterilization units at 22.2%, 18.6%, and 13.3%, respectively [12]. Leapman et al. conducted a life cycle assessment (LCA) of the prostate biopsy pathway. It is estimated that the total greenhouse gas emissions for each procedure amounted to 80.7 kg CO_2_e. The main sources of emissions were the prebiopsy MRI, which accounted for 42.7 kg CO_2_e (52.9% of total emissions), and the biopsy procedure itself, which generated 33.3 kg CO_2_e [13]. Misrai et al. evaluated the carbon footprints and environmental impacts of various MISDs. The study found that the Rezūm device had the highest carbon footprint at 2288 g CO_2_e, followed by the Plasma Loop at 1204 g CO_2_e. Lower emissions were noted for devices like the UroLift (545 g CO_2_e) and Greenlight Laser Fiber (387 g CO_2_e). ITIND and Holmium Laser Fiber (HoLEP) had the smallest footprints at 76 g and 235 g CO_2_e, respectively [14].

**Table 2:** Comparison of environmental impact metrics for single-use and reusable ureteroscopes and cystoscopes across life cycle stages (kg CO_2_ per case)

Study and equipment	Manufacture	Transport	Incineration	Landfill	Solid waste	Sterilization and repackaging	Repair cost (estimated 5 kg CO_2_/repair)	Total
Davis et al. [11]								
LithovueTM single-use ureteroscope	3.83	–	N/S	N/S	0.3	0.3	–	4.43
Olympus URV-F reusable ureteroscope	0.06	–	N/S	N/S	0.005	3.95	0.45	4.47
Hogan et al. [7]								
*aScope 4 Cysto (single-use)*	1.34	0.049	0.61 (0.50–0.64)	0.11	–	0.3	–	2.41 (2.40–2.44)
*Olympus CYF-VA2 cystoscope (reusable)*	0.013	N/A	0.52 (0.51–0.60)	0.22	–	3.5	–	4.23 (4.22–4.24)
P value*	< 0.0001	–	0.3230	< 0.0001		< 0.0001		< 0.0001
Kemble et al. [5]								
*Ambu aScope 4 Cysto (single-use)*	1.59	0.2	N/S	N/S	0.31	0.3	N/A	2.40
*Olympus CYF-V2 (reusable)*	0.0017	Negligible	N/S	N/S	0.000145	0.5	0.02	0.53

#### Waste disposal

Waste disposal comparisons between single-use and reusable cystoscopes reveal varied outcomes. Hogan et al. reported that single-use cystoscopes produce approximately 622 g of waste per procedure, with 27.3% attributed to packaging. While reusable cystoscopes produce greater waste during reprocessing, their cumulative waste output is lower over extended use cycles [7]. In studies by Boucheron et al. further emphasize the environmental trade-offs involved in water consumption for reprocessing reusable cystoscopes (60 L per cycle) [8]. In ureteroscopy, Davis et al. found that single-use devices produce approximately 0.3 kg of solid waste per procedure, with emissions from incineration adding 1.23 kg CO_2_ per kilogram of waste. Reusable ureteroscopes, by contrast, generate less waste overall, estimated at 0.06 kg CO_2_-equivalent per use [11]. The environmental assessment of TURBT procedures by John et al. identified waste from single-use items, including surgical disposables and anesthesia devices, as major contributors to CO_2_e. Additionally, the reprocessing of reusable items produced waste, though to a lesser degree. Waste management processes, including incineration, contributed significantly to overall emissions, underscoring the impact of disposable items in urologic surgery [12].

#### Solid waste produced

Solid waste generated by single-use and reusable cystoscopes illustrates another area of environmental concern. Single-use cystoscopes create approximately 622 g of solid waste per procedure, much of which comes from packaging. Despite generating waste with each reprocessing, reusable cystoscopes, when used over extended cycles, produce a lower cumulative solid waste footprint, as reported by Hogan et al. [7]. In the context of minimally invasive surgical devices (MISDs), Misrai et al. reported that solid waste primarily stems from plastics used in manufacturing and packaging materials, with the carbon footprint of these plastics at 2.38 kg of CO_2_ per kilogram. The study also noted the environmental impact of various MISDs, such as the Rezūm device, Plasma Loop, and UroLift, highlighting the need for sustainable practices in the manufacturing and disposal stages [14].

#### Carbon footprint of waste products

The carbon footprint associated with waste products from urological procedures varies based on the type and frequency of use of single-use versus reusable instruments. In the context of cystoscopy, studies by Hogan and Kemble et al. highlighted emissions from waste incineration as significant contributors to the carbon footprint of single-use devices. Packaging materials, sterilization consumables, and reprocessing waste were primary sources of emissions for reusable devices [5, 7]. In prostate biopsy, a LCA by Leapman et al. estimated the carbon footprint of waste at 1.4 kg CO_2_e per procedure, primarily from medical waste disposal, with additional contributions from incineration and recycling processes. Reprocessing of reusable items contributed minimally to the carbon footprint, though consumable supplies created 1.6 kg of waste per biopsy [13].

Robotic and laparoscopic surgeries, as evaluated by Fuschi et al., underscore the need for energy-efficient practices to reduce the carbon footprint of waste products. The production of laparoscopic instruments led to higher CO_2_e (12,946.73 g) compared to robotic instruments (9506.18 g). Energy consumption for laparoscopic procedures was also greater, with 46,728.24 g CO_2_ compared to 37,807.23 g CO_2_ for robotic surgery. Robotic prostatectomy produced lower emissions than laparoscopic surgery (robotic surgery produces 47,313.41 g CO_2_, in contrast to 59,674.96 g CO_2_ for laparoscopic surgery), yet both generated similar quantities of waste. The higher plastic content in laparoscopic instruments further exacerbates their environmental impact, underscoring the value of adopting robotic technologies with energy-efficient sterilization protocols [15].

## Discussion

Sustainability in urologic practice has gained increasing importance, as healthcare significantly contributes to global carbon emissions and environmental waste, with the sector responsible for 4.4% of global greenhouse gas emissions [16]. Urologists, like other medical professionals, face the challenge of reducing the environmental footprint of their procedures while maintaining high standards of patient care [2, 3]. Given the resource-intensive nature of procedures and technologies involved in urological practices, it is important to recognize the critical role of this aspect. A comparative analysis of single-use versus reusable medical devices in urology, ranging from cystoscopes and ureteroscopes to prostate biopsy tools and minimally invasive surgical devices, reveals the complex interplay between reducing carbon footprints, managing waste, and ensuring proper sterilization [2, 4]. These efforts align with several United Nations Sustainable Development Goals (SDGs), particularly SDG 3 (Good Health and Well-being), by promoting sustainable practices that uphold patient care, SDG 12 (Responsible Consumption and Production), by focusing on reducing CO_2_ emissions, waste disposal, and solid waste production associated with urologic equipment, and SDG 13 (Climate Action), by emphasizing the need to minimize environmental impacts through innovative and eco-friendly solutions [17]. A more balanced, environmentally conscious approach to selecting and using these devices is essential for sustainable urological practice.

One of the primary areas where urologists can have a significant impact on sustainability is within the operating room (OR). The OR is an energy-intensive environment that substantially contributes to a hospital’s overall carbon emissions, consuming three to six times more energy per square meter than other hospital areas [18]. The "Sustainability in the Operating Theatre" guide by the Royal College of Surgeons of England and the Intercollegiate Green Theatre Checklist provide structured, practical recommendations to address these challenges [19, 20]. It is essential to reduce energy consumption and waste within the OR through strategies such as implementing energy-efficient LED lighting, optimizing heating and ventilation systems, and ensuring equipment is powered down when not in use [19, 21].

Efficient sterilization practices, the adoption of reusable instruments, and the optimization of surgical workflows are emphasized as critical measures to minimize the environmental footprint. Waste audits, as highlighted in these resources, have proven effective in identifying areas for improvement, such as reducing unnecessary instruments in surgical trays and promoting the reuse of specific devices [19, 22]. Furthermore, the Green Theatre Checklist advocates for circular economy principles, including the reprocessing of single-use devices and the use of closed-loop recycling systems for surgical waste [20]. By integrating these sustainable practices into routine surgical workflows, urologists and surgical teams can significantly reduce the carbon footprint of the OR while maintaining clinical efficiency and patient care standards.

Endourological instruments including cystoscopes and ureteroscopes play a significant role in urological procedures, making it essential for urologists to understand their usage, sustainability, and environmental impact. Raising awareness about the environmental implications of these devices is vital for promoting sustainable practices in urology. Studies have revealed significant differences in environmental impact between single-use and reusable cystoscopes [5, 7–10]. Single-use cystoscopes like the aScope™ 4 Cysto produce lower CO_2_ emissions per procedure (between 2.06 and 2.41 kg CO_2_) compared to reusable ones like the CYF-VA2, which can generate up to 4.23 kg CO_2_ per use [7, 9]. This is due primarily to the energy-intensive reprocessing required for reusable devices, contributing significantly to emissions. However, it's important to note that single-use cystoscopes also generate a considerable amount of non-recyclable waste (up to 622 g per use) [7]. On the other hand, reusable cystoscopes, despite producing more waste through sterilization consumables, have a lower overall waste footprint per use due to their extended lifespan [7, 8]. In the current literature, there is only one study comparing ureteroscopes and suggests that the reprocessing of reusable devices has a significant impact on the environment. The Olympus URV-F ureteroscope emits 4.47 kg of CO_2_ per procedure, with 3.94 kg of that arising from the energy and water demands of sterilization. While the manufacturing carbon footprint of reusable ureteroscopes is spread out over multiple uses, the emissions from reprocessing can exceed those of single-use alternatives. For example, the single-use Lithovue™ ureteroscope emits slightly less CO_2_ at 4.43 kg per use, but this comes with the drawback of higher solid waste production, once again illustrating the trade-off between emissions reduction and waste management [11].

Sterilization processes are not only a contributor to CO_2_ emissions in cystoscopy and ureteroscopy but also in other urological procedures like TURBT and minimal invasive surgery [12, 14, 15]. In TURBT procedures, 13.3% of the carbon footprint arises from the sterilization of reusable instruments, primarily due to the use of autoclaves powered by steam from gas boilers [12]. These sterilization requirements significantly amplify the environmental impact of reusable instruments, despite their lower waste generation compared to single-use devices. Thus, sterilization practices must be reconsidered, with potential opportunities to optimize and reduce energy and water use through innovations like low-energy sterilization technologies. Beyond sterilization, transitioning from inpatient to day-case TURBT surgeries can result in significant carbon savings. The United Kingdom (UK) National Health Service (NHS) has already reduced its carbon footprint by approximately 2.9 million kg CO_2_ equivalents by increasing day-case surgery rates from 13% in 2013–2014 to 31% in 2021–2022. However, there is still considerable variation in day-case rates across NHS hospitals, and standardizing best practices could lead to further savings of 217,599 kg CO_2_e annually. Additionally, the study found no significant increase in 30-day readmission rates for hospitals with higher day-case surgery rates, confirming the safety of day-case TURBT for patients without complicating conditions. Standardizing best practices across NHS hospitals to increase day-case surgery rates could significantly enhance healthcare efficiency while simultaneously reducing emissions, aligning with broader environmental goals [23].

In robotic and laparoscopic surgeries, it has been showed that robotic prostatectomies have a lower carbon footprint compared to laparoscopic procedures. Robotic surgeries produce approximately 47.3 kg CO_2_ per procedure, whereas laparoscopic surgeries generate about 59.7 kg CO_2_. While robotic systems require less energy, their complex instrumentation and higher plastic content may have more significant long-term environmental impacts [15]. This difference is primarily due to the shorter operative times, reduced energy consumption during hospitalization, and the use of more reusable instruments in robotic surgeries. However, these results focus on operational emissions during the surgical procedure. While the manufacturing of instruments was approximated, the full impact of robot production, maintenance, and disposal was not explicitly detailed in the study. This omission is significant because the production, maintenance, and eventual disposal of robotic systems are resource-intensive and likely contribute significantly to their overall environmental impact. While robotic surgery demonstrates lower emissions during operation, its full life-cycle footprint, including manufacturing and disposal, must be considered to provide a more comprehensive evaluation of its sustainability. There is urgent need for improvements in the production and disposal of materials associated with these procedures, despite the short-term environmental benefits in terms of energy use offered by robotic surgery. The environmental impact of MISDs also points the importance of assessing the entire lifecycle of medical technologies. Plastics, in particular, contribute heavily to the carbon footprint of these devices [14]. A cradle-to-gate analysis shows that the manufacturing and packaging processes account for a large portion of the emissions, urging device manufacturers to explore sustainable materials and packaging solutions.

Leapman et al. conducted a LCA of prostate biopsy procedures and found that each emits 80.7 kg of CO_2_e. Energy consumption, primarily from MRI scans and facility operations, accounts for 57.8% of the emissions. Avoiding unnecessary biopsies or using MRI as a triage tool could lead to significant emissions reductions. Avoiding 100,000 unnecessary biopsies could prevent 8.1 million kg of CO_2_e emissions, equivalent to 4.1 million liters of gasoline. Similarly, using MRI to guide biopsies could save 1.4 million kg CO_2_e per 100,000 patients. The carbon footprint of these procedures varies based on regional energy sources, with countries like Sweden potentially seeing emissions reductions of up to 53% [13]. We should emphasize the importance of improving clinical practices to reduce the environmental impact of urological procedures.

The term ‘carbon footprint' in the medical field is not universally standardized, leading to variations in variables and assessment methodologies. Some studies primarily focus on greenhouse gas emissions related to production and energy consumption, while others incorporate broader environmental impacts, such as waste management and water usage. Our review revealed a significant difference in methodological approaches. Certain studies, such as those comparing single-use versus reusable cystoscopes, primarily assessed the carbon emissions from sterilization and reprocessing. Many studies did not account for indirect emissions related to waste transportation and disposal, which can significantly impact the overall carbon footprint, particularly for single-use devices. The exclusion of these factors led to underestimating the actual environmental burden. Future research should aim for a standardized life-cycle assessment, including direct and indirect carbon footprint contributors.

While carbon footprint is a key metric in environmental sustainability, the overall impact of medical waste generation must also be considered. The accumulation of solid waste, particularly from single-use devices, contributes to environmental degradation through increased landfill burden, incineration emissions, and waste transportation impacts. Furthermore, variations in production techniques, material compositions, and recycling processes over time may influence the reported carbon footprints of medical devices. Older studies may not fully reflect current advancements in sustainable manufacturing and waste management, necessitating cautious interpretation when comparing findings across different periods.

Moreover, it is imperative to address the environmental impact of anesthesiology in urology. General anesthesia, particularly inhaled agents like desflurane, significantly contributes to a hospital's carbon footprint [24, 25]. However, by embracing alternatives such as regional anesthesia and spinal anesthesia, we not only reduce carbon emissions but also improve patient outcomes by minimizing recovery times and hospital stays. The integration of these alternatives in urologic procedures is crucial for minimizing environmental impact and elevating the standard of patient care.

Reducing the carbon footprint in urology can be significantly achieved through day case surgeries, self-removal of catheters, and stent self-removal techniques, as these approaches minimize hospital stays, patient travel, and resource usage. Day-case procedures like HoLEP are safe and effective, even in remote hospital settings, reducing hospital emissions associated with extended patient stays [26]. Similarly, early removal of catheters after surgeries such as robot-assisted radical prostatectomy has demonstrated safety and effectiveness, allowing patients to manage catheter removal at home, thereby reducing unnecessary hospital visits and associated energy consumption [27]. Innovations such as stent on a string (SOS) for post-ureteroscopy patients have also proven safe and effective for self-removal stents at home, minimizing return hospital visits, reducing patient discomfort, and cutting healthcare costs [28, 29]. By adopting these strategies, urology departments can significantly reduce energy use, emissions, and resource wastage while maintaining high standards of patient care and outcomes.

Urology practice extends beyond the operating room. Policies regarding intermittent catheterization also have significant environmental implications. The extensive use of single-use catheters leads to substantial waste production. In the USA alone, this practice results in approximately 85 million pounds of waste annually [30]. While this policy aims to reduce infection risks, there is limited evidence that single-use catheters are more effective than reusable ones at preventing infections [6]. Supporting sustainable catheterization practices could reduce the environmental burden of urologic care.

Telemedicine and other innovations in patient management offer additional opportunities for sustainable urologic practice. Virtual consultations, which became more prevalent during the COVID-19 pandemic, have significantly reduced patient and provider travel, contributing to lower carbon emissions associated with healthcare [31]. Virtual care and decentralized diagnostic tools can help further minimize the carbon footprint of urologic practices.

Finally, efforts to integrate sustainability into clinical guidelines and institutional policies are needed for promoting a greener future in urology. The European Association of Urology has already begun incorporating environmental considerations into its guidelines, emphasizing the need for urologists to be mindful of the ecological impacts of their treatment choices [4]. By prioritizing low-impact interventions, reducing unnecessary tests, and considering the broader environmental effects of medical practices, the urology field can make meaningful contributions to global sustainability goals.

## Conclusion

In conclusion, it is essential to recognize that while single-use devices generally result in lower carbon emissions per procedure, they also contribute significantly to waste generation, posing a serious long-term environmental challenge, especially in terms of incineration and landfill use. Conversely, despite the higher emissions associated with energy-intensive sterilization processes, reusable devices have the potential to minimize waste output over their lifetime. From now on, urological practices must adopt a hybrid approach, improving sterilization processes to reduce energy consumption while encouraging the development of eco-friendly single-use alternatives made from sustainable materials. Achieving sustainability in urology will require collaboration between healthcare providers, device manufacturers, and policymakers to balance the need for patient safety with environmental responsibility.

## Supplementary Information

Below is the link to the electronic supplementary material.

Supplementary file1 (DOCX 18 KB)

## Data Availability

No datasets were generated or analysed during the current study.
