# Supplementary material for: Environmental sustainability in urologic practices: a systematic review
Source: World J Urol. 2025 Mar 6;43(1):152. doi: 10.1007/s00345-025-05522-7 (PMC11885315; doi:10.1007/s00345-025-05522-7)
Supplement: Supplementary file 1 — Supplementary file1 (DOCX 18 KB) [file 345_2025_5522_MOESM1_ESM.docx]

Appendix 1. Search strategy

1. **PubMed**

**((((((((((((((("carbon footprint"[MeSH Terms] OR carbon footprint[Text Word]) OR (sustainability[Text Word])) OR (sustainable surgery[Text Word])) OR (environmental impact[Text Word])) OR (environmental footprint[Text Word])) OR (life cycle[Text Word])) OR (environmental[Text Word])) OR ("refuse disposal"[MeSH Terms])) OR (waste[Text Word])) OR ("carbon dioxide"[MeSH Terms] OR carbon dioxide[Text Word])) OR (CO2 emissions[Text Word])) OR (greenhouse gas[Text Word])) OR ("greenhouse effect"[MeSH Terms] OR greenhouse effect[Text Word]))) AND (urology)) AND (urology OR prostate OR imaging OR endoscopy OR flexible OR reusable OR single-use OR cystoscopy OR ureteroscopy OR catheter)** Sort by: **Publication Date**

("carbon footprint"[MeSH Terms] OR "carbon footprint"[Text Word] OR "sustainability"[Text Word] OR "sustainable surgery"[Text Word] OR "environmental impact"[Text Word] OR ("environmental"[All Fields] AND "footprint"[Text Word]) OR "life cycle"[Text Word] OR "environmental"[Text Word] OR "refuse disposal"[MeSH Terms] OR "waste"[Text Word] OR ("carbon dioxide"[MeSH Terms] OR "carbon dioxide"[Text Word]) OR "co2 emissions"[Text Word] OR "greenhouse gas"[Text Word] OR ("greenhouse effect"[MeSH Terms] OR "greenhouse effect"[Text Word])) AND ("urologie"[All Fields] OR "urology"[MeSH Terms] OR "urology"[All Fields] OR "urology s"[All Fields]) AND ("urologie"[All Fields] OR "urology"[MeSH Terms] OR "urology"[All Fields] OR "urology s"[All Fields] OR ("prostat"[All Fields] OR "prostate"[MeSH Terms] OR "prostate"[All Fields] OR "prostates"[All Fields] OR "prostatic"[All Fields] OR "prostatism"[MeSH Terms] OR "prostatism"[All Fields] OR "prostatitis"[MeSH Terms] OR "prostatitis"[All Fields]) OR ("image"[All Fields] OR "image s"[All Fields] OR "imaged"[All Fields] OR "imager"[All Fields] OR "imager s"[All Fields] OR "imagers"[All Fields] OR "images"[All Fields] OR "imaging"[All Fields] OR "imaging s"[All Fields] OR "imagings"[All Fields]) OR ("endoscopie"[All Fields] OR "endoscopy"[MeSH Terms] OR "endoscopy"[All Fields] OR "endoscopies"[All Fields] OR "endoscopy s"[All Fields]) OR ("flexibilities"[All Fields] OR "flexible"[All Fields] OR "flexibles"[All Fields] OR "pliability"[MeSH Terms] OR "pliability"[All Fields] OR "flexibility"[All Fields]) OR ("reusability"[All Fields] OR "reusable"[All Fields] OR "reusables"[All Fields]) OR "single-use"[All Fields] OR ("cystoscopy"[MeSH Terms] OR "cystoscopy"[All Fields] OR "cystoscopies"[All Fields]) OR ("ureteroscopy"[MeSH Terms] OR "ureteroscopy"[All Fields] OR "ureteroscopies"[All Fields]) OR ("catheter s"[All Fields] OR "catheters"[MeSH Terms] OR "catheters"[All Fields] OR "catheter"[All Fields]))

**Translations**

**urology:** "urologie"[All Fields] OR "urology"[MeSH Terms] OR "urology"[All Fields] OR "urology's"[All Fields]

**urology:** "urologie"[All Fields] OR "urology"[MeSH Terms] OR "urology"[All Fields] OR "urology's"[All Fields]

**prostate:** "prostat"[All Fields] OR "prostate"[MeSH Terms] OR "prostate"[All Fields] OR "prostates"[All Fields] OR "prostatic"[All Fields] OR "prostatism"[MeSH Terms] OR "prostatism"[All Fields] OR "prostatitis"[MeSH Terms] OR "prostatitis"[All Fields]

**imaging:** "image"[All Fields] OR "image's"[All Fields] OR "imaged"[All Fields] OR "imager"[All Fields] OR "imager's"[All Fields] OR "imagers"[All Fields] OR "images"[All Fields] OR "imaging"[All Fields] OR "imaging's"[All Fields] OR "imagings"[All Fields]

**endoscopy:** "endoscopie"[All Fields] OR "endoscopy"[MeSH Terms] OR "endoscopy"[All Fields] OR "endoscopies"[All Fields] OR "endoscopy's"[All Fields]

**flexible:** "flexibilities"[All Fields] OR "flexible"[All Fields] OR "flexibles"[All Fields] OR "pliability"[MeSH Terms] OR "pliability"[All Fields] OR "flexibility"[All Fields]

**reusable:** "reusability"[All Fields] OR "reusable"[All Fields] OR "reusables"[All Fields]

**cystoscopy:** "cystoscopy"[MeSH Terms] OR "cystoscopy"[All Fields] OR "cystoscopies"[All Fields]

**ureteroscopy:** "ureteroscopy"[MeSH Terms] OR "ureteroscopy"[All Fields] OR "ureteroscopies"[All Fields]

**catheter:** "catheter's"[All Fields] OR "catheters"[MeSH Terms] OR "catheters"[All Fields] OR "catheter"[All Fields]

1. **Cochrane**

("carbon footprint" OR sustainability OR "sustainable surgery" OR "environmental impact" OR "environmental footprint" OR "life cycle assessment" OR "environmental burden" OR "waste disposal" OR "carbon dioxide emissions" OR "CO2 emissions" OR "greenhouse gas emissions" OR "greenhouse effect"):ti,ab,kw AND (urology OR urologic OR prostate OR imaging OR endoscopy OR cystoscopy OR ureteroscopy OR "flexible scopes" OR "reusable devices" OR "single-use devices" OR catheter OR "medical waste"):ti,ab,kw

| 1. **Ovid MEDLINE(R) Epub Ahead of Print and In-Process, In-Data-Review & Other Non-Indexed Citations and Daily** |
| --- |
| 1 carbon footprint.tw. 2963 |
| 2 sustainable.tw. 129755 |
| 3 enviromental.tw. 203 |
| 4 environmental footprint.tw. 998 |
| 5 life cycle assessment.tw. 4531 |
| 6 life cycle.tw. 50718 |
| 7 cradle to grave.tw. 342 |
| 8 solid waste.tw. 11192 |
| 9 waste produce.tw. 7 |
| 10 waste disposal.tw. 4115 |
| 11 carbon dioxide emissions.tw. 1350 |
| 12 CO2 emissions.tw. 6241 |
| 13 greenhouse gas.tw. 13491 |
| 14 greenhouse effect.tw. 927 |
| 15 carbon footprint.tiab. 16347 |
| 16 or/1-15 223192 |
| 17 urology.tw. 24686 |
| 18 "endoscopy"/ or endoscopy.tw. 131536 |
| 19 endourology.tw. 918 |
| 20 urologic practice.tw. 340 |
| 21 flexible.tw. 156978 |
| 22 reusable.tw. 10259 |
| 23 ureterosc*.ti,ab,tw. 6741 |
| 24 cystosc*.ti,ab,tw. 12191 |
| 25 catheter*.ti,ab,tw. 240049 |
| 26 or/17-25 566847 |
| 27 16 and 26 3523 |
| 28 limit 27 to (english language and humans) 1040 |
